# The complete chloroplast genome sequences of *Aster souliei* Franch and *Aster tongolensis* Franch (Asteraceae)

**DOI:** 10.1080/23802359.2022.2054375

**Published:** 2022-03-22

**Authors:** Junjun Wang, Riza Zhao, Xueyan Su, Hamamozhi Amu, Zhifeng Zhang

**Affiliations:** School of Pharmacy, Institute of Qinghai-Tibetan Plateau, Southwest Minzu University, Chengdu, China

**Keywords:** *A. souliei*, *A. tongolensis*, phylogenetic analysis, chloroplast genomes

## Abstract

The complete chloroplast genomes of *Aster souliei* Franch and *Aster tongolensis* Franch were reported in this study. The complete chlorogenic genomes of *A. souliei* and *A. tongolensis* were 152,587 bp and 152,571 bp, respectively. The *A. souliei* genome contained two inverted repeat regions (IRs, 25,005 bp), a large single-copy (LSC, 84,409 bp) region, and a small single-copy (SSC, 18,168 bp) region, whereas *A. tongolensis* contained two IRs (25,002 bp), one LSC (84,371 bp), and one SSC (18,196 bp). There were 111 genes in the chloroplast genome of *A. souliei*, consisting of 82 mRNA, 26 tRNA, and three rRNA genes. However, there were 112 genes in the chloroplast genome of *A. tongolensis*, consisting of 83 mRNA, 26 tRNA, and three rRNA genes. Phylogenetic analysis showed that *A. souliei* is in a clade with *A. tongolensis*. This study provides a basis for further phylogenetic studies of *A. souliei* and *A. tongolensis*.

Aster species plants are widely distributed in China. Most of them have been used for thousands of years in Chinese folk medicines with medicinal activities, such as antipyretic, detoxicant, expectorant, and remediable cough properties (Tan et al. 2019). *Aster souliei* Franch and *Aster tongolensis* Franch belong to the genus Aster of Asteraceae (Editorial Committee of Chinese Flora 1985). They are the main sources of ‘Tibetan Aster’ in Tibetan medicines and are clinically used for the treatment of bronchitis, sputum, hemoptysis of tuberculosis, and difficulty in urinating (Shen et al. 2012). Previous studies showed that *A. souliei* contains many kinds of chemical constituents, such as flavonoids, neo-clerodane-type diterpenoids, and triterpenoid saponins (Peng et al. 2015). *A. tongolensis* also contains flavonoids and diterpenoids (Tan et al. 1993). As an important herbal folk medicine, *A. souliei* is becoming increasingly endangered due to its overexploitation and shrinking habitats. However, most previous research has focused on its chemical constituents and medicinal properties (Tian et al. 2012). Few studies have focused on its genetic protection and population diversity. Therefore, we sequenced the chloroplast genome of *A. souliei* and *A. tongolensis* and analyzed the genomic characteristics to provide a scientific basis and reference for further protection and utilization of these medicinal plant resources.

The collection and research of plant materials were carried out in accordance with the guidelines of Southwest Minzu University and national regulations. Two samples and DNA were deposited in the herbarium of this university (contact person: Wang, email: 1299424380@qq.com). *A. souliei* (voucher LY16579) was obtained from Kangding City, Sichuan Province, China (N29°59′20.48″, E101°54.15′79″), and *A. tongolensis* (voucher LY12475) was obtained from Weidi Village, Yajiang County, Sichuan Province, China (N30°02′28.95″, E101°14′14.85″). The experimental process was carried out according to the protocol standard (Illumina Inc., San Diego, CA). A library was constructed, after which the quality of the samples was tested, and then, the library quality was tested. Then, the qualified library was sequenced using Illumina NovaSeq. Sequencing yielded 4.5 G clean data, and SPAdes software was used for genome splicing (Bankevich et al. 2012). CPGAVAS2 was used to annotate the gene and plot it (Linchun et al. 2019). CodonW was used for codon preference analysis. Finally, MISA was used for SSR detection in chloroplasts, and vmatch software was used to find the scattered long repeat fragments in the chloroplast genome (Cock et al. 2010).

The complete genome length of *A. souliei* (OK323961) was 152,587 bp, including two inverted repeats (IRs, 25,005 bp), a large single-copy (LSC, 84,409 bp) region, and a small single-copy (SSC, 18,168 bp) region. The complete genome of *A. tongolensis* (OK323962) was 152,571 bp in length. There were two IRs (25,002 bp), one LSC (84,371 bp), and one SSC (18,196 bp). The GC contents of the *A. souliei* and *A. tongolensis* genomes were 37.31% and 37.32%, respectively, which were lower than the 43.03% and 43.03% in the IR region but higher than the 35.23% and 35.21% in the LSC region and 31.31% and 31.30% in the SSC region, respectively.

There were 111 genes in the chloroplast genome of *A. souliei*, consisting of 82 mRNA, 26 tRNA, and three rRNA genes. The sequence coding lengths for amino acids in the protein (CDS), rRNA gene, and tRNA gene were 71,946, 8842, and 1968 bp, respectively. There were 112 genes in the chloroplast genome of *A. tongolensis*, consisting of 83 mRNA, 26 tRNA, and three rRNA genes. The sequence coding lengths for amino acids in the protein, rRNA gene, and tRNA gene were 77,520, 8842, and 1968 bp, respectively.

Phylogenetic analysis to infer the position of *A. souliei* and *A. tongolensis* in the family of Asteraceae was based on the chloroplast genomes of *A. souliei* and *A. tongolensis* evaluated by the researcher and nine other chloroplast genomes published on the National Center for Biotechnology Information (NCBI) website (Figure 1). The genome sequences of *Artemisia argyi* and *Artemisia annua* were used as an outgroup. The sequences in this study were aligned using Mega-X version 10.2.6 software with 1000 bootstrap replicates (Kumar et al. 2018). The results showed that *A. souliei* and *A. tongolensis* were in the same branch as homologous species, with a bootstrap value of 96%. This paper provides useful data for studying the phylogenetic relationship between *A. souliei* and *A. tongolensis* in the Asteraceae family and provides a basis for studying their genetic diversity.

**Figure 1.**
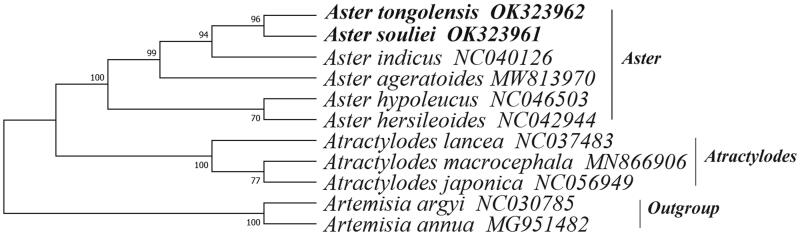
The maximum-likelihood tree of *Aster tongolensis* and *Aster souliei* and other Asteraceae species based on whole chloroplast genome sequences.

## Authors contributions

Junjun Wang performed the data analysis and wrote the manuscript; Riza Zhao contributed to the conception of the study; Xueyan Su performed the experiment; Hamamozhi Amu contributed significantly to analysis and manuscript preparation; Zhifeng Zhang made the critical revisions of intellectual content; and all authors have agreed to be accountable for all aspects of the work.

## Data Availability

The genome sequence data that support the findings of this study are openly available in GenBank from NCBI at https://www.ncbi.nlm.nih.gov/ under accession nos. OK323962 and OK323961. The associated BioProject, SRA, and Bio-Sample numbers are PRJNA765189-PRJNA765149, SRR16296519-SRR16293895, and SAMN21547077-SAMN21545269, respectively.
